# EPMA summit 2014 under the auspices of the presidency of Italy in the EU: professional statements

**DOI:** 10.1186/s13167-015-0026-2

**Published:** 2015-02-24

**Authors:** Olga Golubnitschaja, Vincenzo Costigliola, EPMA

**Affiliations:** Department of Radiology, Rheinische Friedrich-Wilhelms-Universität Bonn, Sigmund-Freud-Str. 25, 53105 Bonn, Germany; The European Association for Predictive, Preventive and Personalised Medicine, 1150 Brussels, Belgium

**Keywords:** Predictive preventive personalised medicine, Healthcare, Innovation, Non-communicable diseases, Rare diseases, Research, Technology, Education, Implementation, Expert recommendation

## Abstract

Over the next 10–20 years, a pessimistic prognosis considers pandemic scenario for type 2 diabetes mellitus, neurodegenerative disorders and some types of cancer followed by the economic disaster of healthcare systems in a global scale. Well-recognised deficits of currently provided medical services result from the delayed ‘disease care’. Herewith EPMA releases the long-term strategies for the effective promotion of predictive, preventive and personalised medicine (PPPM) considered as the medicine of the future. Under the EPMA-umbrella, an international forum of currently 45 countries is actively contributing to the development and implementation of the innovative PPPM concepts. EPMA is open for collaboration with other leading European and global professional networks relevant for the effective promotion of PPPM in sciences and practical implementation.

## Unpredictable, unpreventable and impersonal medicine: deficits of current healthcare

Over the next 10–20 years, a pessimistic prognosis considers pandemic scenario for an increasing number of chronic diseases, such as type 2 diabetes mellitus, neurodegenerative disorders, some types of cardiovascular diseases and cancer followed by the economic disaster of healthcare systems in a global scale. Well-recognised deficits of current healthcare is the delayed ‘disease care’ with all negative consequences such as the low efficacy of medical services, decreased quality of the patient life, frequent hospitalisation and increasing economical and societal burden.

‘We have the knowledge for disaster reduction, what we need is the action’ [1].

## PPPM—medicine of the future and evidence-based platform to advance healthcare

**Figure 1:**
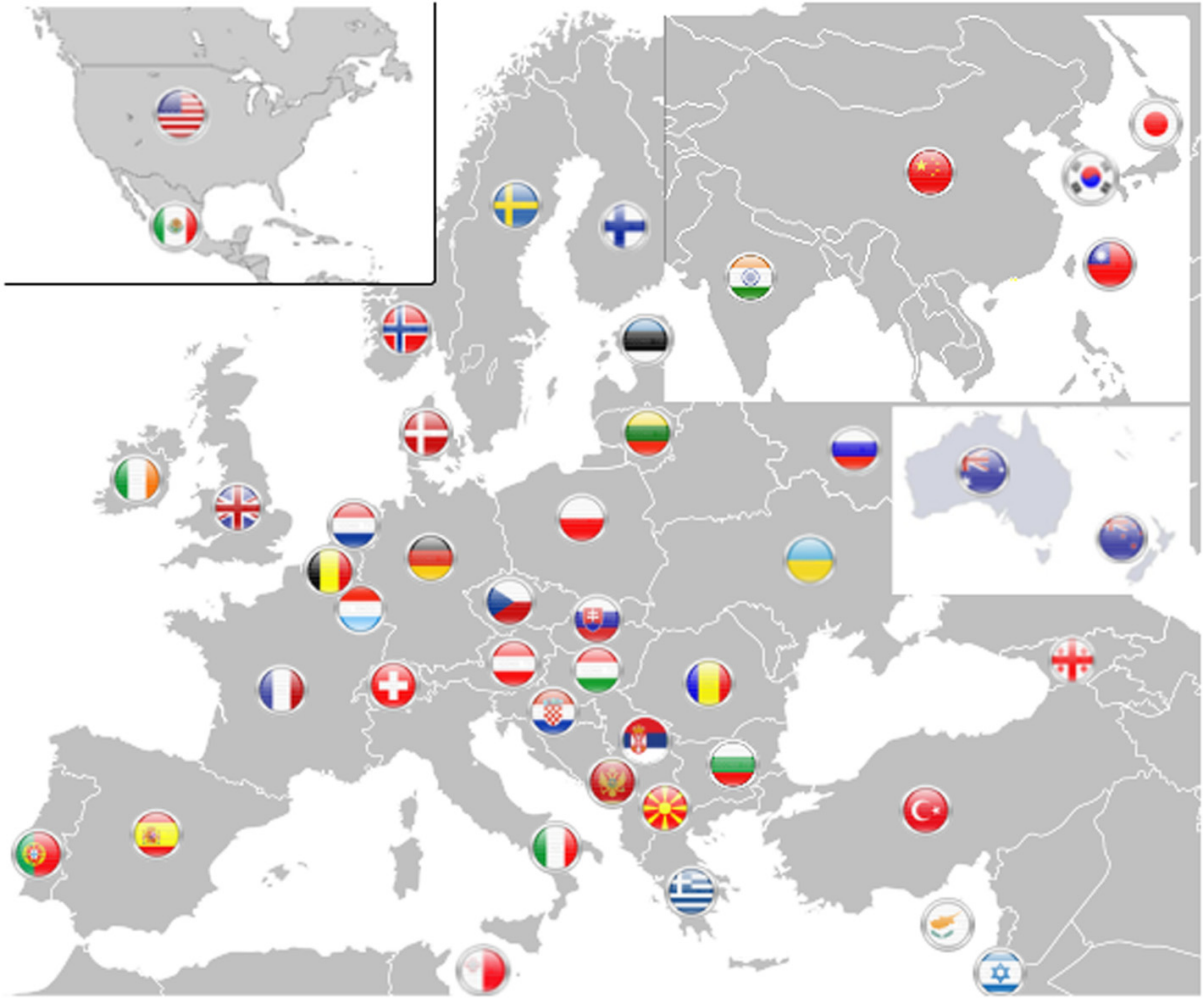
**International EPMA platform: worldwide 45 countries actively contribute to the creation and implementation of innovative PPPM concepts in medical sciences, technologies and healthcare.** The group of the National Representatives (National EPMA BOARDs) is the key element in the structure of the association which consolidates and coordinates the EPMA-related activities at the national level closely working with all PPPM-related national institutions, units and groups such as Federations of Patients, Universities, research units, state and private hospitals, insurance, industrial groups, policy-makers, etc. More information—please see the EPMA website www.epmanet.eu.

**Figure 2:**
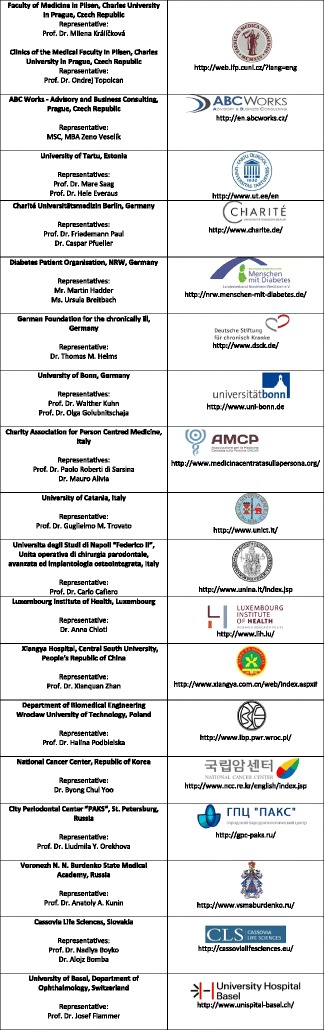
**EPMA institutional members create a nucleus of the expert working groups in the association consolidating professionals for effective networking in PPPM, promoting high-quality topic related research and contributing to creation of guidelines in European healthcare.** EPMA institutional members are the long-term partners participating in the thematic international projects and dedicated European programmes such as ‘Horizon 2020’.
- Industrial Advisory Board (Figure 3)

Advanced healthcare promotes the paradigm change from delayed interventional to predictive medicine tailored to the person, from reactive to preventive medicine and from disease to wellness. Predictive, preventive and personalised medicine (PPPM) is the new integrative concept in healthcare sector that enables to predict individual predisposition before onset of the disease, to provide targeted preventive measures and create personalised treatment algorithms tailored to the person. Integrative approach by PPPM is considered as the medicine of the future. The cost-effective management of health promotion and disease care implements the innovation by PPPM in modernisation of medical services. Being at the forefront of the global efforts, the European Association for Predictive, Preventive and Personalised Medicine (EPMA, http://www.epmanet.eu/) promotes the integrative concept of PPPM amongst healthcare stakeholders, governmental institutions, educators, funding bodies, patient organisations and in the public domain [2]. Under the EPMA-umbrella, an international forum of currently 45 countries is actively contributing to the development and implementation of the innovative PPPM concepts (see Figure 1) worldwide. The main structures altogether create a consolidated scientific, technological and educational platform and elaborate the long-term strategies of the association, namely:

- Institutional members (Figure 2)

**Figure 3:**
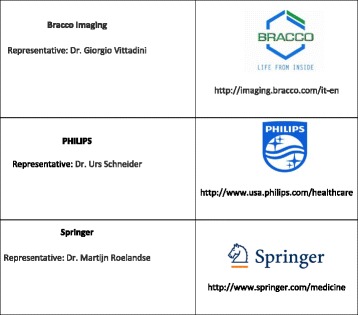
**EPMA Industrial Advisory Board (IAB) is the main structure in the association bridging research and implementation activities in PPPM.** IAB provides professional advices considering innovative concepts, practical needs and related strategies. Members of the IAB are the long-term partners invited by the association in consensus with thoroughly elaborated criteria for a partnership which the mission is to effectively promote the field of PPPM.
- Specialised professional sections (see below)
- Group of the section-responsible Associate Editors (see below)
- Young professionals section, EPMA-YPS (Figure 4)

**Figure 4:**
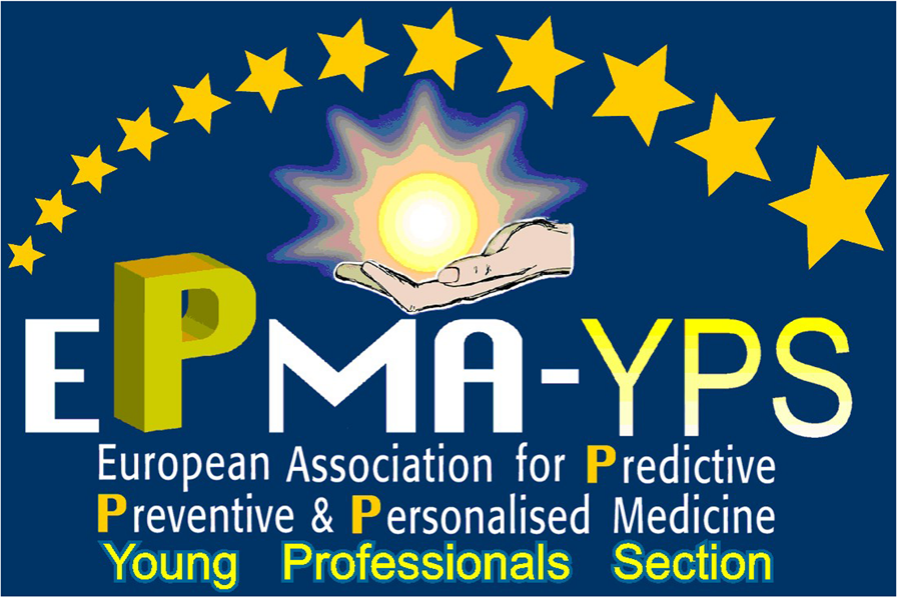
**The specialised ‘Young Professional Section’ aims at the promotion of education and career of young professionals in the field of PPPM; for more information—please see** **www.epmanet.eu** **.**
- EPMA/Springer book series ‘Advances in PPPM’

## Long-term strategies of the European Association for Predictive, Preventive and Personalised Medicine

*Prioritised medical fields* listed and shortly described below have been approved as being of *strategic interests of the association*. The study was approved by the EPMA BOARD of Directors, Group of Associate editors responsible for individual sections, EPMA Institutional members and EPMA Industrial Advisory Board. Consequent realisation will be organised by the EPMA using following instruments:

- ➢ multilateral professional and international collaborations,
- ➢ excellent research activities,
- ➢ high-quality publications (*The EPMA Journal*, EPMA Book-Series and other instruments),
- ➢ scientific projects responding to the calls of the ‘Horizon 2020’ (and other appropriate national and international programmes), international meetings (EPMA Summits/World Congresses and other appropriate forums) [3],
- ➢ implementation of the evidence-based research achievements for an effective advancing of healthcare by innovative medical services (creation and qualification of PPPM centres of excellence),
- ➢ PPPM-related education for both professionals and general population,
- ➢ effective career promotion of young professionals creating a highly qualified professional elite for the realisation of PPPM objectives via new generations of experts in the EU and global scale.

*Specialised professional sections*

1. **Healthcare and Medical Services** (responsible editor: Vincenzo Costigliola, Belgium)
  In healthcare, realisation of optimistic versus pessimistic prognosis depends on current innovations in diagnostic and cost-effective treatment approaches widely adopted in clinical practice. How do we estimate the overall impact of personalised medicine and adopt innovative approaches in healthcare systems, whilst promoting predictive diagnostics, targeted preventive measures and individualised patient treatment on a global scale? EPMA has created a scientific forum for professionals to discuss this topic. The main objectives of these efforts are to mark stakeholders in the field, to consolidate professional groups and to elaborate expert recommendations of how to optimise these approaches for the patient [4,5].
2. **Cardiovascular Disease** (responsible editors: Hiroyasu Iso, Japan, and Hans-Peter Brunner-La Rocca, The Netherlands)
  The field of cardiovascular disease (CVD) is one of the major targets for PPPM. There is a large body of evidence concerning cardiovascular risk factors and preventive strategies at both population and individual levels but also chronic disease stages that are not adequately addressed because they do not follow the PPPM principles. This section focuses on the broad aspects of basic, clinical and epidemiological studies. The promotion of PPPM in the treatment of CVD is a global health issue, since the health burden from CVD is currently the most severe in most developed countries and is rapidly increasing in most developing countries. It is therefore of the utmost importance to exchange on a global-level scientific insights, knowledge and skills for risk prediction of cardiovascular disease, and to share and adopt various experience for its prevention and the development of personalised treatment approaches [6,7].
3. **Cancer** (responsible editors: Dominic Desiderio, USA, and Byong Chul Yoo, South Korea)
  Cancer research shows that more data are needed on the molecular basis (genes, transcriptomes, proteomes, PTMs) of each cancer; molecular changes during development of a cancer; and treatment. A new paradigm in medicine—predictive, preventive, and personalised medicine—clarifies mechanisms, yields an accurate diagnosis and leads to effective treatment. We have a unique molecular environment; cancer develops differently in each person; and we respond individually to drugs. One size does not fit all. PPPM synergy in a cancer study accelerates cancer research and treatment [8,9].
4. **Neurological, Neuropsychiatric and Neurodegenerative Diseases (NNND)** (responsible editors: Friedemann Paul, Germany & Silvia Mandel, Israel)
  Neuropsychological/neuropsychiatric ailments and progressive neurodegeneration are common features of the majority of socially and economically devastating disorders and diseases with multifactorial physical and cognitive disability. NNND have pathologies of a multifactorial nature involving interplay of epigenetic and environmental risk factors. Insights into molecular pathomechanisms will facilitate the creation of the most effective targeted protective strategies and individualised treatment before pathologies manifest. Multifunctional (multi-drug) therapies should be tailored to individual multi-aetiological aspects of the disorders, in order to advance healthcare of patient cohorts. Particular emphasis should be placed on primary prevention by the identification of predisposed individuals early on in life, followed by treatments tailored to the person, which need regulations supported by innovative reimbursement programmes. This strategy creates a robust platform for the cost-effective medicine of the future [10-13].
5. **Diabetes** (responsible editors: Mahmood S. Mozaffari, USA, and Babak Baban, USA)
  The worldwide increase in the incidence and prevalence of diabetes mellitus (DM) continues to exert alarming burden on healthcare systems. The consequent cost impact poses a major challenge for both developed and developing countries/economies. These prevailing conditions provide the rationale for the concept of PPPM: the prediction of persons at risk should help devise strategies for treatments tailored to the individual and to prevent target organ complications of DM, thereby, reducing morbidity and mortality and associated costs. EPMA emphasises the need to address the integrative approach for diabetes care and innovative science focused on the patient [14].
6. **Rare Diseases (RDs)** (responsible editor: Meral Özgüc, Turkey)
  RD is defined as being a rare one, if it affects less than 5 persons per 10,000 individuals. Although each individual RD is rare, altogether they are 5,000–8,000 distinct RDs affecting many millions of people worldwide. Currently, it is estimated that in Europe alone, there are at least 30 million of RD patients. Almost 80% of RDs have a genetic origin with symptoms appearing in prenatal and early postnatal periods. Currently, there are no appropriate treatment approaches for most of the RDs. The only reasonable approach seems to be a development of methods for early diagnosis of RDs that might lead to the creation of the optimal care management saving lives and improving life quality within the patient cohort. The improvement in RDs healthcare is initiated by legislations in EU and the USA to create an integrative medical approach for RDs. How the emerging paradigm of PPPM may improve healthcare in RDs? Due to the molecular background of most RD pathologies, it is expected that the multimodal approach (*omics, pharmacogenetics, medical imaging, etc.) with high multidisciplinarity of professionals should be instrumental for the ‘personalisation’ to diagnose individual RDs, to create effective preventive measures and to develop targeted therapies—the integrative medical approach by PPPM [15].
7. **Traditional Complementary and Alternative Medicine** (responsible editors: Paolo Roberti di Sarsina, Italy, and Wei Wang, China)
  This section provides a platform for innovative strategies in science and healthcare demonstrating how traditional, complementary and alternative medicine (TCAM) can enrich the paradigm of predictive, preventive and personalised medicine. Evidence-based TCAM scientific approaches should demonstrate high levels of person-centred and participatory medicine, functional links between TCAM, predictive diagnostics, targeted prevention and individualised treatments in healthcare education and clinical practice. Topics should explore tailoring care by investigating and treating the person as a physical, psychological and spiritual unicum living in dynamic interaction with nature and society. Individualise the therapeutic process by renewing the caregiver-person relationship with trust, compassion and respect for personal choices. Explore innovative research methods to evaluate TCAM and other salutogenetic interventions as a form of preventive medicine empowering communities and individuals [16-18].
8. **Pain Management** (responsible editor: Rostyslav Bubnov, Ukraine)
  This section demonstrates the highest levels of multidisciplinarity and benefits from excellent competencies of all medical fields (including neuroscience, psychology, imaging, sport medicine and TCAM) and complex technological instruments (including hybrid technologies). The main focus is a deep diagnostics followed by creating individualised treatment algorithms. The research and review articles are invited, dealing with pain-relevant issues within integrated vision of PPPM. This includes topics relevant animal models, translation research, novel physiological, safe and personalised therapies developed for minimally interventional pain management and physiotherapy, such as dry needling, neuromodulation, etc. Regenerative therapy, guided by advanced imaging techniques, using 3D modelling, prospects for development of rapid prototyping, robotics, smart prosthetics, etc. are the desirable focuses of innovation of the pain management. This section aims to encourage publications relevant to practitioners and researchers in a variety of medical fields, covers topics of musculoskeletal disorders, rheumatologic, orthopaedic, neurologic conditions involving acute and chronic pain, peripheral nervous systems, rehabilitation and military medicine. Further, research to predictive, preventive and prognostic pain management relevant biomarkers is considered by the section. Articles dedicated to self-assessment as well as focused on creation of smart questionnaires are welcome. Prevention of a wide spectrum of collateral diseases (NDD, metabolic syndrome, cancer) linked to the pain management may be considered in the context of the improved healthcare policy and economic benefits of the societies [19].
9. **Dentistry** (responsible editor: Mahmood S. Mozaffari, USA)
  Oral/dental health contributes to the overall health and well-being of everybody. A growing body of evidence demonstrates that the manifested dental and oral pathologies are linked to the increased risk of various diseases including heart and lung disease, vascular pathologies, stroke, diabetes mellitus, neurological disorders, pre-term birth and even some types of cancer, amongst others. Further, certain oral symptoms are considered as the early indicator of a spectrum of the mental disorders such as anorexia, bulimia, anxiety and depression. On the other hand, dental diseases themselves may be caused by acute and chronic systemic disorders such as diabetes mellitus. Whilst an association between oral/dental diseases and systemic disorders is well established, the cause-and-effect relationships in these conditions are poorly understood, an investigation of which is a prerequisite for predictive, preventive and personalised dental medicine [20-22].
10. **Transplantation** (responsible editor: Hele Everaus, Estonia)
  Cell and organ transplantation has obtained extremely important role in the treatment of several severe and fatal diseases. Long waiting lists of patients worldwide reflect major problems and current deficits in transplantation medicine. Prediction and personalisation in transplantation are essential that require an identification of individual pre- and post-transplantation biomarker panels allowing better donor/recipient matching and assessment of individual risks. Improved donor-recipient matching, person-centred immunosuppressive regimens, individual risk assessment for chronic allograft damage and prediction of graft accommodation—altogether may lead to substantially increased allograft survival and decreased patient morbidity, therefore, advancing this medical area in a global scale. Currently, very vivid research on stem cells creates new perspectives in the area. An integration of basic sciences at molecular level, technological advances and clinical sciences is crucial to progress the area and satisfy the unmet patient needs in the field. Further, medical ethics, smart political regulations and economy have remarkable impacts on advances in transplantation and regenerative medicine, in general.
11. **Clinical Nutrition, Environment and Health Psychology** (responsible editors: Guglielmo M. Trovato, Italy, and Niva Shapira, Israel)
  The growing body of knowledge on nutrition requires the dissemination of well-elaborated information within the scientific and public domains. This section focuses on integrative medical approaches for individualised nutrition by means of predictive, preventive and personalised medicine. It aims to create professional opinions and to enhance and develop knowledge and skills taking into account evidence-based scientific achievements in the fields of epidemiology, healthy lifestyle, optimised nutrition, food science/technology/culture, medical ethics and cost-effective healthcare and environmental and affordable strategies. We welcome innovative approaches for nutrition in health and disease, which can then be used to build up expert recommendations and standardisation. Our goal is to produce an evidence-based consensus for sustainable guidelines in predictive medicine together with targeted prevention in healthy individuals, persons at-risk and stratified patient groups with manifest diseases and provide advice to stratified patient groups, institutions, food producers and marketing experts [23,24], also using the assessment and intervention tools of clinical nutrition and health psychology. Health and disease main determinants are genetic, environmental and behavioural, and each component is merged and interacts with the others. Environment is a still neglected topic in PPPM; nonetheless, geography, climate, anthropic modification, urban, rural and occupational, agriculture and fishing are all subsets that should be considered, along with societal issues and political and economical implication, for increasing the possibility of successful outcomes in health promotion. A greater attention is warranted within the comprehensive objectives of research, also in other field, with a perspective of predictive, preventive and personalised medicine.
12. **Body Culture and Sports Medicine (BCSP)** (responsible editors: Rostyslav Bubnov, Ukraine, and Halina Podbielska, Poland)
  This section aims to understand how basic and clinical research can be applied to personalised and preventive strategies as set out by the framework of EPMA. BCSP covers a wide spectrum of topics, ranging from but not limited to exercise, healthy lifestyle, personalised sleep algorithms, homoeopathy, physical therapy, rehabilitation and others. Disease-modifying effects that manifest in disease states, such as hypertension, hypercholesterolemia, obesity, neurodegenerative disorders, etc., are in the focus of this section. High-quality research based on measurable effects (including clinical criteria, imaging and molecular biomarkers) that are associated with modifiable factors (nutrients, physical activity, lifestyle, etc.) is promoted by this section, with a particular focus on research for individually tailored interventions [25-27].
13. **Translational Medicine** (responsible editors: Anna Chioti, Luxembourg, and Mikhail Paltsev, Russia)
  This section aims to bridge excellent achievements in basic and applicative sciences with an effective implementation of innovative approaches in PPPM to advanced healthcare services to the patients. With the increasingly complex relationship between basic research and clinical application, there is a pressing need to bridge the translational gap from bench to clinic using integrative methods. Topics should explore advances in pathophysiology, disease modelling, biomarker discovery and validation, novel diagnostics (including companion diagnostics) in personalised medicine which should help translate knowledge from studies at the bench side to care at the bed side. Clinical trials and public health studies generating data allow to develop new research hypotheses with a potential to unlock new mechanisms. New approaches in drug development, including data management, bioinformatics and systems biomedicine, allow to address the whole continuum of translation research in genomic medicine: from gene discovery to health application, to evidence-based guideline, to health practice and finally to health impact [28,29].
14. **Information and Communication Technologies (ICT)** (responsible editors: Heinz U. Lemke, Germany, and Michael Legg, Australia)
  PPPM proposes innovative strategies towards an ICT-based holistic presentation of the individual patient and corresponding medical processes/procedures. These strategies imply a redesign of healthcare activities within a given domain of medical discourse, such as cardiovascular, neurological, diabetic or oncologic disorders. ICT systems support provided by a medical information and model management system-like architecture, which includes a number of carefully selected diagnostic and therapeutic core functionalities, is the prerequisite for an effective PPPM. With a holistic presentation of a specific patient based on appropriate mathematical modelling methods, such as probabilistic relational models and process models as well as advanced ICT-enabling tools, the practice of medicine will be substantially transformed towards model-based medical evidence, providing transparency of clinical situations, processes and decisions for patient and physician. ICT approaches may result in profound and cost-effective modernisation of healthcare. The beneficiaries of these transforming methods and technologies will include patients, healthcare providers and society at large [30,31].
15. **Innovative Technologies (IT)** (responsible editors: Kurt Krapfenbauer, Austria, and Xianquan Zhan, China)
  This section promotes any technological innovation which may lead to advanced healthcare services such as non-invasive, early and predictive diagnostics, targeted prevention and treatments tailored to the person. IT can be used for identification, as well as characterisation and validation of clinically relevant biomarkers. For example, medical imaging, sub-cellular imaging, *omics (genomics, transciptomics, proteomics, metabolomics, etc.) and hybrid technologies developed can be used to identify potential biomarker patterns at several molecular levels. Based on IT such as highly sensitive molecular chip technologies, the biomarker research has faced many significant challenges. The use of biomarkers and novel technologies to investigate clinically relevant information allows proper patient stratification into therapeutic groups and improved therapy outcomes. If detected well in time, smart molecular alterations can optimise therapy outcome in specific therapeutic groups. Integrating this information allows selection of personalised targeted treatment regimes, saving unnecessary drug toxicity and decreasing morbidity [32-34].
16. **Pharmacogenetics** (responsible editors: Godfrey Grech, Malta, and Romano Danesi, Italy)
  Currently, the use of genetic information to treat patients is still in its early stages, with some clear successes mostly in the oncology and infectious disease therapeutic areas. Some successful examples include the targeting of tailored pharmaceuticals developed for the treatment of patients with a particular disease subtype or according to a specific genetic makeup pertaining to the drug’s mode of action. In other examples, genetic information is being used to help determine the effective and safe dose of specific pharmaceuticals. However, implementation of this pharmacogenetic knowledge to the clinic has proven to be challenging and to require collaboration between the various stakeholders throughout the discovery, development and validation stages so as to ensure the utility of actionable genetic testing in a cost-effective manner. The challenges of poly-pharmacy are of particular importance to be considered by individualised targeting. Targeted therapy and reliable prediction of expected outcomes offer patients access to better healthcare management, by way of identifying the therapies effective for the relevant patient group, avoiding prescription of unnecessary treatment and reducing the likelihood of developing adverse drug reactions [35].
17. **Laboratory Medicine** (responsible editors: Marko Kapalla, Slovak Republic, and Elvar Theodorsson, Sweden)
  The section is focused on predictive and prognostic laboratory tests considered as the robust platform for more precise diagnosis, targeted prevention and treatments tailored to the person. Current deficits in medical services such as delayed intervention, untargeted medication, overdosed patients and ineffective treatments require the role of laboratory shifted from the ‘passive performing’ to the ‘active advising’ one. Recommendations by the laboratory to assist clinical practice are highly desirable. This assistance ranges from advising on the necessity for additional tests to the dynamic analysis of the targets. Additional tests should be considered from the viewpoint of their reasonability, in order to reach an accurate and realistic health-related data interpretation for the individual. The analysis of dynamic changes of the target is essential to evaluate potential health impacts such as an individual predisposition to the disease and/or a predictive diagnosis before a clinical manifestation of symptoms. Laboratory value-added investigation and interpretation should be obligatory in creating an advanced functional relationship between laboratory medicine and clinicians together as the responsible decision-makers [36-38].
18. **Biobanking and Biopreservation** (responsible editor: Judita Kinkorova, Czech Republic)
  Internationally valid biobanking is currently an ongoing process, facing major viability challenges which are in the focus of this section. As for individual types of biological material (tissue samples, blood samples, DNA, RNA, proteins, metabolites, etc.), unified/standardised instructions and legal approaches should be validated how samples may be collected, stored, retrieved, shared and tested. The analytical quality of collections, storage condition and donation of samples to a biobank require a strict control both at national and international levels. Disease-focused collections require acquired samples to be retrospectively valid for a development of novel biomarkers and novel drugs/treatments. For a disease-specific biobanking, patients should be stratified with immaculate record-keeping, to enable to draw conclusions on the new markers. The functional link to the reliable clinical data and interpretation is crucial for the utility of any biobank [36,39].
19. **Biomarkers** (responsible editors: Suzanne Hagan, UK, and Byong Chul Yoo, South Korea)
  If biomarkers are discovered, are they applicable to diagnostics, treatment targets/monitoring or both? Is a biomarker defined on a molecular basis? Does one biomarker unambiguously define corresponding pathology? Does a new diagnostic approach apply to groups of risks and targeted prevention? Is a multi-level biomarker panel applicable/available to secure a precise diagnose and therapy targeting? This section is in a mission to respond to the above questions [40-42].
20. **Medical Chemistry** (responsible editor: Jorge F. J. Coelho, Portugal)
  This section involves a multidisciplinary approach that ranges from the application of innovative active therapeutic medicines to advanced methods for controlled delivery. Particular attention is paid to outstanding developments involving structure—biological activity relationships in different areas of interest for this topic, such as: stem cells; rational drug design, new (co)polymers; creation of tailor-made drug delivery systems; incorporation of target molecules to the polymeric structures; encapsulation of approved drugs with the polymers; preparation and characterisation of nanoparticles for *in vivo* diagnostics and treatment; and evaluation and validation of new systems *in vitro* and *in vivo* studies [43-46].
21. **Design** (responsible editors: Antony Kent, UK, and Guglielmo M. Trovato, Italy)
  The specific challenge for multidisciplinary communication is the design of media to facilitate effective interaction amongst professional groups in PPPM. These groups currently ‘speak different languages’ which reinforce each group’s professional perspective and frequently underestimates the added value of the transfer of products between disciplines. The specific output of this designing activity is the so-called ‘professional interactome’ and the representation of complex networks of information. The interactome represents the most optimal model of healthcare organisation designed specifically for the implementation of an effective interaction amongst professional groups in PPPM that creates the main focus of this section. Recognising the need for engagement and to enhance an patient’s ease of access to, and usability of, services and products, the design principles of envisioning and empathy, ergonomics and prototyping will be applied to the conceptualisation and development of personalised services (service and experience design) and personal medical devices (product design). Since ‘style in the life’ is one of the most important factors affecting health status and its perception, particularly in youngsters, design in the wider sense of the concept and, namely, fashion’s trends strongly influences media, health-related behaviours, body size, shape and appearance perception. For these reasons, multidimensional assessment and intervention aimed to favourable modifications of lifestyles should include the awareness that urban, home and work design, even clothing and fashion and related industries, can be addressed to the enhancement of PPPM intervention [47].
22. **Education** (responsible editor: Ondrej Topolcan, Czech Republic)
  This section aims at creating new didactic and educational measures, including e-learning tools, conducting important information for all PPPM relevant professional groups (medical doctors, students, nurses, etc.), patients and their family members as well as individuals as risk and targeted groups in populations, introducing innovative concepts of PPPM and shifting the paradigm from delayed reactive care, to predictive and preventive medical care. It will distinctively draw on current multimedia design practices (see the above paragraph 21) to create attractive and motivational learning materials as well as communications targeted to key groups. The ultimate goal of this activity is to support the creation of a new generation of professionals in medicine who will be able to implement a holistic approach to patient care that recognises the complexity and individuality of any organism instead of treating the patient as a disaggregated ‘pool of organs’ [48,49].
23. **Business Models** (responsible editor: Frank Heemskerk, Belgium)
  Poor economy of healthcare systems and delivery is in the focus of this section. Across Europe, there is a great diversity of systems, payment and reimbursement schemes. This imposes a highly fragmented market (market access is governed by various public and private organisations) which raises the efforts (seeking recognition, market authorisation and reimbursement in 28 different countries and respective bodies) and the costs (all different bureaucratic schemes) considerably. On the one hand, there is a need for policy dialogue to achieve some harmonisation in rules and delivery but also in access and reimbursement for patients across Europe. On the other hand, there is also a great need for more advanced business models (what is the offer?, who benefits?, who pays?) to motivate all stakeholders for better scientific achievements, more effective implementation, improved medical services and interest in the general population to follow the strategies of predictive and preventive medicine for cost-effective healthcare. In the light of economic strain and with ageing populations, this innovation in the healthcare systems is critical to keep the high quality of healthcare we have in Europe affordable and sustainable [50,51].
24. **PPPM Centres—the nucleus for advanced healthcare** (EPMA working group: Vincenzo Costigliola, Belgium; Josef Flammer, Switzerland; Mikhail Paltsev, Russia; Marko Kapalla, Slovak Republic; Juraj Kuban, Slovak Republic; Kurt Krapfenbauer, Austria; Olga Golubnitschaja, Germany)
  Successful PPPM implementation requires an unprecedented level of collaboration amongst all stakeholders, long-term multidisciplinary professional partnerships including public-private ones, robust juristic platform and smart political regulations. It is important that future developments are focused on the integration of all elements of PPPM. Innovative PPPM centres are focused on designing and conducting a new culture in healthcare: high level of multidisciplinarity, innovation and professional education, well-met patient needs, cost-effective economy of healthcare, etc.

## Collaboration with other professional groups leading dedicated medical fields relevant for PPPM

Below listed professional networks have clearly nominated areas of professional interests tightly linked to the EPMA objectives. Corresponding agreements have been signed creating a robust platform for successful bilateral collaborations in a long-term way.

**European Medical Association** (EMA, www.emanet.org); EPMA and EMA support each other developing complementary activities in disseminating PPPM relevant information, providing high-quality professional education, performing daily work with patient organisations and implementing innovative approaches to advance medical services in Europe.

**European Society of Radiology** (ESR, www.myesr.org); EPMA and ESR have proclaimed the long-term partnership and mutual support in effective promotion of common efforts in the predictive diagnostics, targeted preventive measures and personalised patient treatment in the European healthcare and in global scale. The development of professional synergies between EPMA and ESR are expected to lead to much progress in the PPPM generally and, in particular, strengthen the role of the issue-related European networks. Considering these clear strategic benefits, both EPMA and ESR decided to collaborate considering following items:

- creation of expert recommendations for advanced healthcare
- collaborative promotion of innovative medical fields and the philosophy of integrative medicine
- collaborative development of scientific projects dedicated to translational medicine and practical application of innovative technologies in routine medical practice
- networking of institutional members in Europe and worldwide
- mutual enrichment of the long-term strategies and outlook in PPPM by concepts development and regular publishing of position articles in both journals—*The EPMA Journal* and *European Radiology*
- mutual support of educational and training programmes by inviting experts and providing expertise related to diverse branches of PPPM
- creation of innovative didactic materials for PPPM-related educational and training programmes
- collaborative development of granting strategies and synergies in setup of innovative projects related to PPPM, e.g. in the European ‘Horizon 2020’
- creation of specialised EPMA/ESR workshops at the regular EPMA World Congresses and ESR Congresses
- Mutual support in publicity (website, public relation, etc.).

**European Federation of Clinical Chemistry and Laboratory Medicine** (EFLM, www.efcclm.org); EFLM provides European leadership in clinical chemistry and laboratory medicine. Both EPMA and EFLM consider the integrative approach by PPPM as the medicine of the future. In furtherance of their mutual interest in advancing the healthcare paradigm from reactive to predictive, preventive and personalised medicine and in laboratory medicine as the integrating element in PPPM, the two organisations recognise the added value of working together and initiate the establishment of the joint Working Group (WG). The strategic document about EPMA/EFLM professional synergies recently has been published in *The EPMA Journal* to define clear priorities of the collaboration [36].

**European Society of Predictive Medicine** (EUSPM, www.euspm.org); EPMA and EUSPM are joining the efforts for the predictive diagnostics, targeted preventive measures and personalised patient treatment in the European healthcare. Professional synergies are developed, and bilateral support is provided concerning all scientific, granting, publication, practical application and any other network-related activities.

**European Depression Association** (EDA, www.europeandepressionday.com); EDA is interested in professional collaboration with the EPMA focusing specifically on predictive diagnostics, targeted prevention and advanced person-centred approaches for the most optimal treatment of depression as the stratified patient cohort.

**European, Middle-Eastern and African Society for Biopreservation and Biobanking** (ESBB, www.esbb.org); Designed as being reliable for advanced healthcare services, biobanking considers the interests of all parties involved in the research and business. International biobanking is in a focus of both EPMA and ESBB creating the solutions to the below listed unmet needs, namely

- How can local and international barriers be broken down to unify international biopreservation technologies, sample collections and databases?
- Who carries the role to promote in the process of biobanking internationalisation?
- Who should cover the costs of the internationally accessible systems?
- Who is allowed to legally use these international biobanks once they are created?
- Who could be nominated as the end user of the data collected?

EPMA is open for collaboration with other leading European and global professional networks relevant for the effective promotion of PPPM.
